# The impact of gestational diabetes mellitus on maternal-fetal pregnancy outcomes and fetal growth: a multicenter longitudinal cohort study

**DOI:** 10.3389/fped.2025.1592550

**Published:** 2025-05-20

**Authors:** Mi Su, Yangyang Wang, Qinqin Yuan, Dongmei Tang, Yu Lu, Xixi Wu, Wen Xiong, Yalan Li, Tianjiao Liu, Siyuan Zeng, Sumei Wei

**Affiliations:** ^1^ Chengdu Women’s and Children’s Central Hospital, School of Medicine, University of Electronic Science and Technology of China, Chengdu, China; ^2^ Department of Gynaecology and Obstetrics, Chengdu Xinjin District Maternal and Child Health Care Hospital, Chengdu, China; ^3^ The Fourth People’s Hospital of Chengdu, School of Medicine, University of Electronic Science and Technology of China, Chengdu, China; ^4^ Key Laboratory of Birth Defects and Related Diseases of Women and Children (Sichuan University), Ministry of Education, West China Second Hospital, Sichuan University, Chengdu, China

**Keywords:** cohort study, food allergies, gestational diabetes mellitus, infant growth, maternal-neonatal

## Abstract

**Objective:**

To investigate the impact of gestational diabetes mellitus (GDM) on maternal and neonatal pregnancy outcomes and fetal growth patterns.

**Methods:**

A cohort of 418 pregnant women was analyzed, comprising 203 with normal glucose tolerance and 215 diagnosed with GDM. Key maternal factors, including age, pre-pregnancy body mass index (BMI), gestational weight gain, and gestational hypertension, were assessed for their association with infant growth and food allergy outcomes. At six months of corrected gestational age, weight-for-age z-scores (WAZ) and food allergy incidence were compared between the two groups. Binary logistic regression and linear regression analyses were performed to identify significant predictors of these outcomes.

**Results:**

Infants born to mothers with GDM exhibited significantly higher WAZ scores (*p* = 0.026) and an increased neonatal susceptibility to food allergies (*p* = 0.043) compared to those born to mothers with normal glucose tolerance. Maternal factors such as advanced age, higher pre-pregnancy BMI, gestational hypertension, and twin pregnancy were identified as key risk factors for GDM. Additionally, preterm birth, birth weight, and parental history of allergies were independently associated with the development of food allergies in infants.

**Conclusion:**

GDM exerts a notable influence on infant growth trajectories and elevates the risk of food allergies. Effective glycemic management during pregnancy, early monitoring of infant development, and targeted interventions addressing risk factors such as preterm birth and parental allergy history are critical for mitigating long-term health risks in children exposed to GDM *in utero*. Further research is warranted to explore the underlying mechanisms and potential preventive strategies for this at-risk population.

## Introduction

Gestational diabetes mellitus (GDM), a prevalent metabolic disorder during pregnancy, is characterized by glucose intolerance that is first recognized or diagnosed during gestation (1). The global incidence of GDM has risen significantly over the past few decades, paralleling the increase in obesity, sedentary lifestyles, and advanced maternal age (2, 3). GDM is associated with a spectrum of adverse maternal and neonatal outcomes, including hypertensive disorders of pregnancy, macrosomia, preterm birth, and an elevated risk of cesarean delivery (4, 5). Moreover, its impact often extends beyond delivery, with both mothers and their offspring facing elevated risks of long-term metabolic complications such as type 2 diabetes and obesity (6).

In addition to these well-established outcomes, growing evidence suggests that GDM may influence fetal growth trajectories and early developmental processes. Intrauterine exposure to maternal hyperglycemia can lead to impaired placental function, altered nutrient transport, and an inflammatory intrauterine environment, which in turn may affect fetal growth patterns and organ development (7, 8). While macrosomia is a frequent outcome, some fetuses may exhibit growth restriction, depending on the degree and timing of glycemic control during pregnancy. These alterations in the intrauterine environment have been linked to potential long-term consequences for neurodevelopment, including increased risks of cognitive and behavioral disorders in childhood (9, 10).

Recent studies also indicate that maternal hyperglycemia may adversely impact neonatal immune maturation. Mechanisms such as epigenetic modification, systemic inflammation, and changes in the maternal and neonatal gut microbiota have been proposed to mediate this effect (11). These changes may increase the susceptibility of the offspring to immune-related disorders, including food allergies, which have been rising in prevalence globally (12).

Despite progress in GDM screening and treatment, the interplay between maternal hyperglycemia, fetal growth, and immune development remains incompletely understood. This study aims to comprehensively assess the impact of GDM on maternal and neonatal pregnancy outcomes, fetal growth, and early immune development, using data from a large-scale multicenter longitudinal cohort. Findings from this study may help address important gaps in current knowledge and inform both clinical practice and public health interventions.

## Material and methods

### Study design and participants

This clinical study aimed to investigate the impact of gestational diabetes mellitus (GDM) on maternal and neonatal outcomes and fetal growth. It was conducted as a retrospective, multicenter longitudinal cohort study between March 2021 and June 2023 (ChiCTR2100052428), representing a completed clinical phase with postnatal follow-up. Pregnant women in their second trimester were recruited from two tertiary hospitals: West China Second University Hospital and Chengdu Women's and Children's Central Hospital. Participants were eligible if they had no preexisting diabetes or autoimmune diseases and provided written informed consent for both maternal and infant follow-up, including postnatal assessments at 6 months of corrected infant age. The study was approved by the Ethics Committee of Chengdu Women's and Children's Central Hospital (Approval No. 2021107) and conducted in compliance with the Declaration of Helsinki. GDM diagnosis was made between 24 and 28 gestational weeks using a 75-g oral glucose tolerance test (OGTT), and participants were categorized into either the GDM group or the normal glucose tolerance (NGT) group based on OGTT results.

### GDM diagnostic criteria

GDM was diagnosed between 24 and 28 weeks of gestation using a standard 75-g OGTT, in accordance with the International Association of Diabetes and Pregnancy Study Groups (IADPSG) criteria (13). GDM was defined if one or more of the following thresholds were met: fasting plasma glucose ≥5.1 mmol/L, 1-h glucose ≥10.0 mmol/L, or 2-h glucose ≥8.5 mmol/L.

### Data collection

Maternal demographic information, medical history, and obstetric outcomes were collected from hospital records. Pregnancy outcomes, including preterm birth, mode of delivery, neonatal birth weight, and Apgar scores, were recorded. Using pediatric health data at six months of corrected age for neonates to collect breastfeeding practices, infant feeding patterns, and parental allergy history.

### Infant growth assessment and skin prick test

Infant growth was assessed at 6 month of corrected age using weight, length, and head circumference measurements. Growth parameters were standardized using World Health Organization (WHO) growth standards.

The skin prick test (SPT) was conducted at 6 months to assess food allergy sensitization. The panel of allergens included cow's milk proteins (casein and β-lactoglobulin), egg white (ovalbumin), egg yolk, wheat, soybean, peanut, tree nuts (including walnut, cashew, and almond), fish (cod and salmon), and shellfish (shrimp and crab). These allergens were selected based on their known prevalence and clinical relevance in early childhood food allergy. A positive SPT result was defined as a wheal diameter ≥3 mm greater than the negative control.

### Statistical analysis

*A priori* sample size calculation was performed based on estimates from previous cohort studies examining the effects of GDM on infant development. Assuming a power of 80% and an alpha level of 0.05 to detect meaningful differences in infant outcomes, a minimum of 158 participants per group was required. To account for an anticipated 10% attrition rate, the target enrollment was set at 174 participants per group.

Descriptive statistics were used to summarize maternal and neonatal characteristics, with means (±SD) or medians (IQR) reported for continuous variables and frequencies (%) for categorical variables. Comparisons between the GDM and control groups were conducted using Student's *t*-tests or corrected Student's *t*-tests for continuous variables and chi-square tests for categorical variables. Multivariable logistic regression models were used to examine the association between GDM and adverse pregnancy outcomes, adjusting for potential confounders such as maternal age, body mass index, and parity. Generalized linear models were applied to assess the impact of GDM on infant growth parameters. Logistic regression was also employed to investigate the relationship between GDM and food allergy sensitization, as determined by SPT results. A two-tailed *p*-value <0.05 was considered statistically significant. Analyses were conducted using SPSS version 26.0 (IBM Corp., Armonk, NY, USA).

## Results

**Figure 1:**
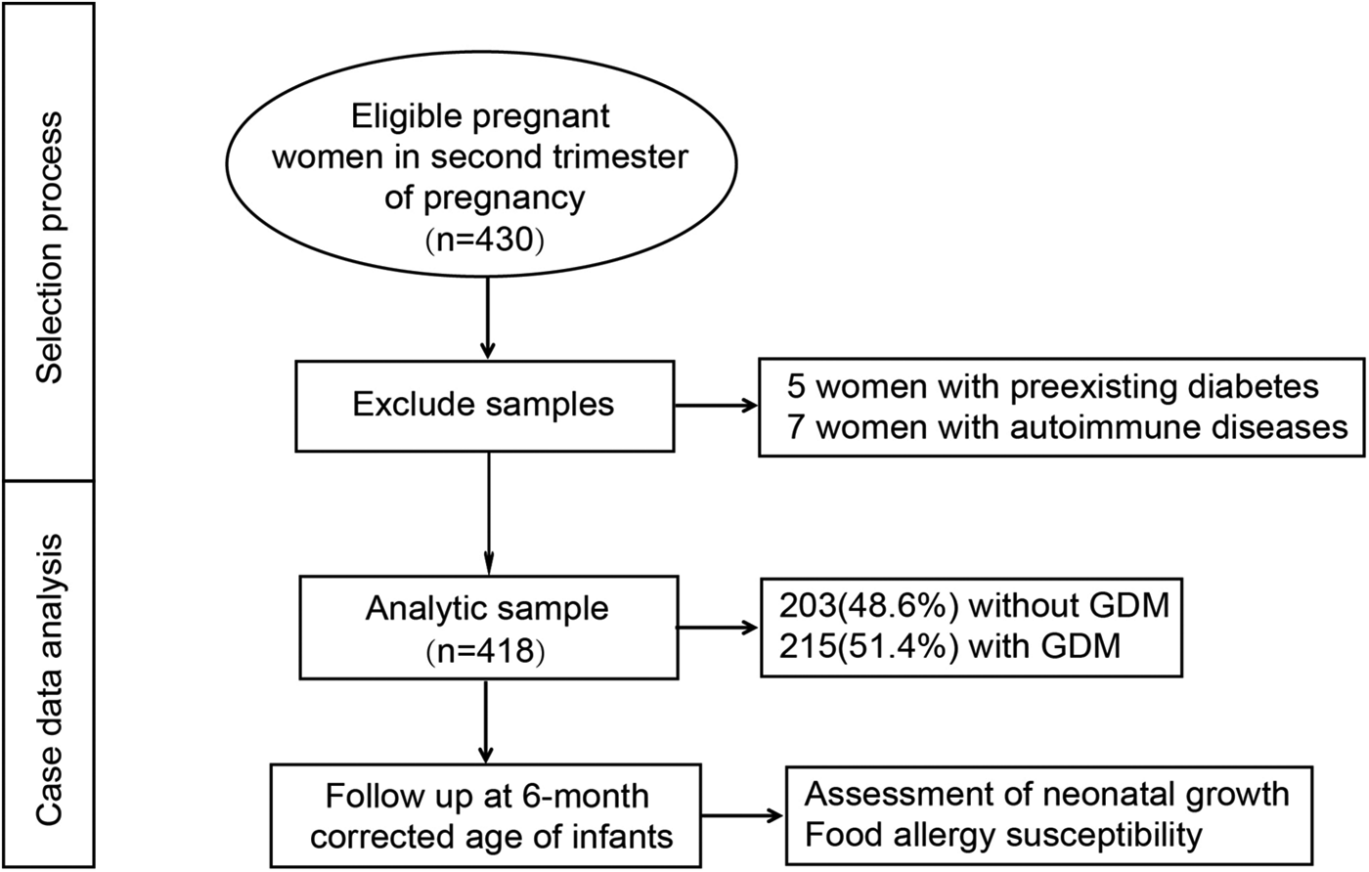
The selection process for this study.

A total of 430 eligible pregnant women were initially enrolled in the study. Of these, five cases were excluded due to preexisting diabetes, and seven cases were excluded due to autoimmune diseases. Ultimately, 418 participants were included in the final analysis, comprising 203 women in the normal glucose tolerance (NGT) group (48.6%) and 215 women in the GDM group (51.4%). The selection process for the study population is illustrated in Figure 1. At the time of enrollment, the mean maternal age was 29.57 ± 4.42 years. The mean gestational age at delivery was 39.04 ± 1.78 weeks, and the mean neonatal birth weight was 3,185.35 ± 424.42 g. Notably, 29 cases (6.9%) were twin pregnancies, and 20 cases (4.8%) resulted from assisted reproductive technology (ART), as detailed in Table 1.

**Table 1:** Description of the maternal and neonatal characteristics.

Variables	Total
Mothers	418
Maternal age (year)	29.57 ± 4.42
Pre-pregnancy BMI (kg/m^2^)	22.26 ± 2.63
Mode of conception	
Natural conception	398 (95.2%)
Assisted reproductive technology	20 (4.8%)
Type of pregnancy	
Singleton pregnancy	389 (93.1%)
Twin pregnancy	29 (6.9%)
Gestational weight gain (kg)	13.03 ± 3.11
Gestational age (week)	39.04 ± 1.78
Preterm birth	16 (3.8%)
Type of pregnant women by OGTT	
Normal glucose tolerance women	203 (48.6%)
Gestational diabetes mellitus women	215 (51.4%)
Infants	447
Gender (male)	226 (50.6%)
Birthweight (g)	3,185.35 ± 424.42
Neonatal susceptibility to food allergies	42 (9.4%)

BMI, body mass index, OGTT, oral glucose tolerance test.

Univariate analysis revealed that, compared to pregnant women with normal glucose tolerance, those in the GDM group exhibited significantly higher pre-pregnancy BMI and neonatal birth weight (*p* < 0.05). Additionally, the GDM group had a higher risk of gestational hypertensive disorders (GHD), fetal growth restriction (FGR), and fetal asphyxia (*p* < 0.05) (Table 2).

**Table 2:** Description of the maternal and neonatal characteristics by pregnant women types.

Variables	Normal glucose tolerance group	Gestational diabetes mellitus group	*P*-value
Mothers	*N* = 203	*N* = 215	
Age (year)	29.25 ± 4.23	29.87 ± 4.59	0.151^a^
Pre-pregnancy BMI (kg/m^2^)	21.70 ± 2.44	22.79 ± 2.80	<0.001^a^
Mode of conception			
Natural conception	194 (95.6%)	204 (94.9%)	0.744^b^
Assisted reproductive technology	9 (4.4%)	11 (5.1%)	
Gestational weight gain (kg)	12.87 ± 2.83	13.18 ± 3.36	0.307^a^
Gestational age (week)	39.17 ± 1.33	38.91 ± 2.12	0.132^a^
Preterm birth	5 (2.5%)	11 (5.1%)	0.158^b^
Gravidity	2.01 ± 0.51	1.95 ± 0.46	0.208^c^
Parity	0.92 ± 0.18	0.89 ± 0.15	0.066^c^
Pregnancy-induced illness			
ICP	13 (6.4%)	16 (7.4%)	0.676^b^
GHD	8 (3.9%)	23 (10.7%)	0.008^b^
FGR	7 (3.4%)	18 (8.4%)	0.034^b^
Twin pregnancy	9 (4.4%)	17 (7.9%)	0.142^b^
Mode of delivery (Cesarean section)	87 (42.9%)	97 (45.1%)	0.642^b^
24-h bleeding volume after delivery	434.93 ± 123.32	460.93 ± 188.40	0.094^a^
Infants	*n* = 212	*n* = 235	
Gender (male)	104 (49.1%)	122 (51.9%)	0.546^b^
Birthweight (g)	3,090.45 ± 396.48	3,270.97 ± 448.13	<0.001^a^
Neonatal susceptibility to food allergies	2 (0.9%)	10 (3.8%)	0.039^c^

BMI, body mass index, ICP, intrahepatic cholestasis of pregnancy, GHD, gestational hypertension disorder, FGR, fetal growth restriction.

^a^ Average and standard deviation. Student's *t*-test.

^b^ Number (percentage). Chi-squared test.

^c^ Number (percentage). Fisher exact test.

**Figure 2:**
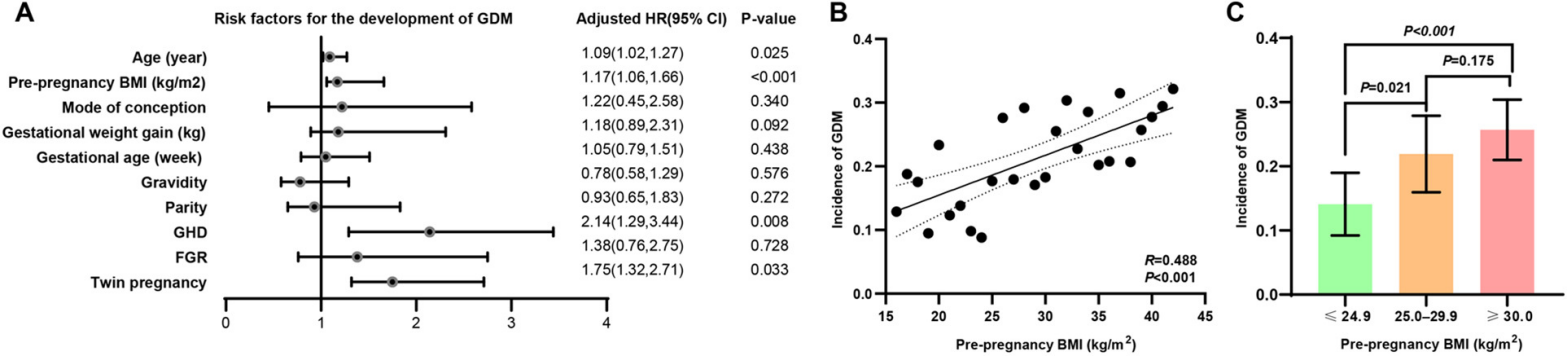
The impact of maternal characteristics on the risk of GDM. **(A)** After adjusting for covariates such as mode of conception, gestational weight gain, gestational age, gravidity, parity, and FGR, the results of binary logistic regression analysis indicated that maternal age (β = 1.09, 95% CI: 1.02–1.27, *p* = 0.025), pre-pregnancy BMI (β = 1.17, 95% CI: 1.06–1.66, *p* < 0.001), GHD (β = 2.14, 95% CI: 1.29–3.44, *p* = 0.008), and twin pregnancy (β = 1.75, 95% CI: 1.32–2.71, *p* = 0.033) were independent risk factors for GDM. **(B)** Further linear regression analysis demonstrated a positive correlation between pre-pregnancy BMI and the incidence of GDM (R = 0.488, *p* < 0.001). **(C)** Subgroup analysis revealed a stepwise increase in the incidence of GDM across BMI categories: normal/underweight women (BMI ≤24.9), overweight women (BMI 25.0–29.9), and obese women (BMI ≥30). The incidence of GDM was significantly higher in overweight compared to normal/underweight women (BMI ≤24.9 vs. 25.0–29.9, *p* = 0.021), with a trend toward higher incidence in obese compared to overweight women (BMI 25.0–29.9 vs. ≥30, *p* = 0.175).

After adjusting for covariates such as mode of conception, gestational weight gain, gestational age, gravidity, parity, and FGR, the results of binary logistic regression analysis indicated that maternal age (OR = 1.09, 95% CI: 1.02–1.27, *p* = 0.025), pre-pregnancy BMI (OR = 1.17, 95% CI: 1.06–1.66, *p* < 0.001), GHD (OR = 2.14, 95% CI: 1.29–3.44, *p* = 0.008), and twin pregnancy (OR = 1.75, 95% CI: 1.32–2.71, *p* = 0.033) were independent risk factors for GDM (Figure 2A). Specifically, for each additional year of maternal age, the risk of developing GDM increased by 9%; for each additional unit of pre-pregnancy BMI, the risk of GDM increased by 17%. Moreover, GHD and twin pregnancy were statistically associated with an increased risk of GDM, with adjusted OR of 1.14 and 0.75, respectively. However, given the relatively small number of cases for these variables—particularly twin pregnancies (*n* = 29)—these associations should be interpreted with caution due to the wide confidence intervals and potential instability of the estimates (Figure 2A).

Given that pre-pregnancy BMI demonstrated the most significant association with the occurrence of GDM in the multiple linear regression analysis, a further linear correlation analysis was conducted to examine the relationship between BMI and the incidence of GDM. The result demonstrated a positive correlation between pre-pregnancy BMI and the incidence of GDM (R = 0.488, *p* < 0.001) (Figure 2B). Subgroup analysis revealed a stepwise increase in the incidence of GDM across pre-pregnancy BMI categories: normal/underweight women (BMI ≤24.9), overweight women (BMI 25.0–29.9), and obese women (BMI ≥30). The incidence of GDM was significantly higher in overweight compared to normal/underweight women (BMI ≤24.9 vs. 25.0–29.9, *p* = 0.021), with a trend toward higher incidence in obese compared to overweight women (BMI 25.0–29.9 vs. ≥30, *p* = 0.175) (Figure 2C).

At the corrected gestational age of six months, infants in the GDM group demonstrated a significantly higher weight-for-age z-score (WAZ) compared to the control group (*p* = 0.003). Additionally, the neonatal susceptibility to food allergies was notably higher in the GDM group (*p* = 0.025) (Table 3).

**Table 3:** Description of the neonatal growth and susceptibility to food allergies by pregnant women types.

Variables	Normal glucose tolerance group *N* = 212	Gestational diabetes mellitus group *N* = 235	*P*-value
WHZ	0.37 ± 0.81	0.48 ± 0.73	0.134^a^
WAZ	0.35 ± 0.62	0.57 ± 0.93	0.003^a^
HAZ	0.41 ± 0.65	0.52 ± 0.70	0.086^a^
BAZ	0.39 ± 0.76	0.48 ± 0.89	0.250^a^
Allergic to one or more food	13 (6.1%)	29 (12.3%)	0.025^b^
Number of food allergic kinds	1.47 ± 0.56	1.58 ± 0.65	0.057^a^

WHZ, *z*-score for weight-for-height; WAZ, *z*-score for weight-for-age; HAZ, *z*-score for height-for-age; BAZ, *z*-score for BMI-for-age.

^a^ Average and standard deviation. Student's *t*-test.

^b^ Number (percentage). Chi-square test.

After adjusting for covariates such as infant gender, breastfeeding status, and household pets, binary logistic regression analysis identified preterm birth (OR = 2.55, 95% CI: 1.43–4.15, *p* = 0.032), birth weight (OR = 0.33, 95% CI: 0.09–0.54, *p* = 0.045), maternal history of GDM (OR = 1.83, 95% CI: 1.05–3.65, *p* = 0.048), and parental allergy history (OR = 1.79, 95% CI: 1.16–2.83, *p* < 0.001) as independent factors associated with the risk of food allergies in infants. Specifically, for every 100 g increase in neonatal birth weight, the risk of food allergies decreased by 6.7%. Conversely, preterm birth, maternal history of GDM and parental allergy history increased the risk of food allergies by 155%, 83% and 79%, respectively (Table 4).

**Table 4:** Association between neonatal susceptibility to food allergies and maternal and infant characteristics.

Variables	Exp(B)	95% CI	*P*-value
Preterm birth	2.55	1.43–4.15	0.032
Gender	1.40	0.65–2.07	0.483
Birthweight (kg)	0.33	0.09–0.54	0.045
Maternal history of GDM	1.83	0.97–3.65	0.048
Parental allergy history	1.79	1.16–2.83	<0.001
Non exclusive breastfeeding	1.55	0.88–2.91	0.726
Pet at home	1.32	0.96–2.35	0.563

Exp(B), odds ratio calculated as the exponential of the regression coefficient B, GDM, gestational diabetes mellitus.

## Discussion

This study provides a comprehensive examination of the impact of GDM on infant development, particularly in relation to growth patterns and the risk of food allergies. The findings underscore the significant influence of maternal metabolic conditions during pregnancy on offspring health, with implications for clinical care and public health strategies.

### Implications for infant growth trajectories

Infants born to mothers with GDM exhibited altered growth trajectories, as evidenced by higher weight-for-age z-scores at six months of corrected gestational age. This observation reflects the influence of intrauterine metabolic conditions on postnatal growth dynamics. Maternal hyperglycemia likely leads to increased transplacental glucose transfer, stimulating fetal insulin production, a potent growth factor (4, 14). These growth patterns may predispose affected infants to accelerated weight gain, which could have long-term health implications, including an increased risk of obesity and metabolic disorders (15). Clinically, these findings highlight the importance of early monitoring and potential growth interventions in infants exposed to GDM, as such measures may help mitigate future health risks.

### The role of GDM in immune development

The increased neonatal susceptibility to food allergies observed in infants born to mothers with GDM is a notable finding with significant public health implications. While the precise pathways remain to be fully elucidated, this association suggests that maternal GDM may predispose offspring to immune dysregulation, potentially due to systemic effects of maternal hyperglycemia (16, 17). Clinical recognition of this increased risk is critical, particularly in guiding dietary management and early allergen introduction in high-risk infants.

This study also identified other maternal and perinatal factors, including preterm birth, birth weight, and parental allergy history, as independent determinants of food allergy risk in infants. These findings underscore the multifactorial nature of allergic disease development and suggest potential opportunities for risk stratification and targeted intervention. The association between preterm birth and increased food allergy risk is consistent with evidence that premature infants have underdeveloped immune and gastrointestinal systems (18). This highlights the need for enhanced nutritional and immunological support for this population to reduce allergy susceptibility. The inverse relationship between birth weight and allergy risk suggests that adequate fetal growth plays a protective role in immune system development (19, 20). This finding reinforces the importance of promoting optimal fetal growth during pregnancy to support immune health. A strong familial predisposition to allergies emphasizes the interplay between genetic and environmental factors (21). Parental allergy history should be considered a critical component in assessing and managing the allergy risk in infants, guiding preventive measures such as tailored feeding practices and allergen exposure protocols.

The findings of this study have direct implications for maternal and child health care. For women with GDM, achieving optimal glycemic control during pregnancy should be prioritized not only to reduce perinatal complications but also to minimize long-term risks to their offspring (22, 23). Postnatal care strategies should include growth monitoring and early screening for allergic conditions, particularly in infants with additional risk factors such as preterm birth or a family history of allergies. Additionally, promoting exclusive breastfeeding and supporting dietary diversification during infancy may serve as effective measures to enhance immune resilience.

While our findings support an association between maternal GDM and increased neonatal susceptibility to food allergies, it is important to acknowledge that not all studies have reached similar conclusions. For example, a recent population-based birth cohort study did not find a significant relationship between maternal metabolic conditions and the risk of food allergies in offspring (24). This discrepancy may reflect differences in population genetics, environmental exposures, or diagnostic criteria used across studies. In our study, food allergy sensitization was assessed via standardized SPT, which is widely accepted as a reliable method for evaluating IgE-mediated allergic responses in infancy. However, it is important to recognize that SPT results reflect sensitization rather than clinical allergy, and oral food challenges—the gold standard for diagnosis—were not performed due to ethical and logistical constraints. Therefore, while the observed associations are meaningful, they should be interpreted cautiously.

The increasing prevalence of GDM worldwide necessitates heightened awareness of its potential impacts on offspring health. These findings highlight the need for public health initiatives that integrate maternal health management into broader child health strategies. Educational campaigns aimed at reducing modifiable risk factors, such as maternal obesity and poor glycemic control, could have far-reaching benefits for both mothers and their children.

Moreover, our findings underscore the relevance of additional maternal and perinatal variables in influencing infant outcomes. For instance, preterm birth emerged as an independent risk factor for food allergy sensitization, aligning with the hypothesis that immune system immaturity in preterm infants may increase allergen susceptibility. Similarly, low birth weight, which may indicate suboptimal intrauterine nutrition or placental dysfunction, was inversely associated with food allergy risk. Parental allergy history, a proxy for genetic predisposition, remained one of the strongest predictors. These results reinforce the multifactorial etiology of allergic diseases and highlight the need for integrative risk assessment models that account for both metabolic and immunologic pathways in the maternal-fetal interface.

This study's strengths include its robust statistical analysis and the consideration of multiple confounding factors. However, several limitations should be acknowledged. The study's sample size, while sufficient to detect significant associations, may limit the generalizability of the findings to broader populations. The small sample sizes for subgroups may have led to wide confidence intervals and increased susceptibility to variability, which could affect the robustness of the findings. Additionally, the reliance on a single-center cohort highlights the need for multi-center studies to confirm these results. Finally, while the associations observed are compelling, causality cannot be definitively established, underscoring the need for further research. Future research should focus on the long-term outcomes of infants born to mothers with GDM, particularly regarding metabolic and immune health. Expanding the scope of research to include diverse populations and larger cohorts will enhance the generalizability of findings. Additionally, exploring preventive and therapeutic strategies, such as tailored dietary interventions for mothers and infants, could provide actionable solutions to mitigate the risks associated with GDM.

## Conclusions

This study highlights the significant impact of GDM on infant development, particularly in terms of altered growth patterns and an increased susceptibility of food allergies. Infants born to mothers with GDM demonstrated higher weight-for-age z-scores and a greater likelihood of developing food allergies compared to those born to mothers without GDM. Key factors influencing these outcomes included preterm birth, birth weight, and parental allergy history. These findings emphasize the importance of effective maternal glycemic control during pregnancy and early monitoring of at-risk infants to mitigate long-term health risks. Further research is needed to explore the long-term effects and potential interventions for children exposed to GDM.

## Data Availability

The original contributions presented in the study are included in the article/Supplementary Material, further inquiries can be directed to the corresponding authors.

## References

[1] Yen FS Wei JC Wu YL Lo YR Chen CM Hwu CM Impact of family income on the development of gestational diabetes mellitus and the associated birth outcomes: a nationwide study. J Diabetes Investig. (2024). 10.1111/jdi.14288 PMC11693567 39540712

[2] Candido R Toffoli B Manfredi G Turisani A Delfauro V Petrucco A Retrospective cohort study on treatment outcomes of early vs late onset gestational diabetes mellitus. Acta Diabetol. (2024). 10.1007/s00592-024-02405-y 39527298

[3] Chen Y Wang H Yang Y Li J Luo T Wei H Latent profile analysis of sleep quality in pregnant women with gestational diabetes mellitus and its influencing factors. West J Nurs Res. (2024):1939459241296728. 10.1177/01939459241296728 39520211

[4] Chiu HY Chen HH Wang CW Lu H Wu CH Yang CC The risks of emergency C-section, infant health conditions and postpartum complications in Taiwanese primiparous women with gestational diabetes mellitus: a propensity matched cohort study. Taiwan J Obstet Gynecol. (2024) 63(6):880–6. 10.1016/j.tjog.2024.01.039 39481996

[5] El Seifi OS Younis FE Ibrahim Y Begum SB Ahmed SF Zayed ES Telemedicine and gestational diabetes mellitus: systematic review and meta-analysis. Cureus. (2024) 16(10):e71907. 10.7759/cureus.71907 39564055 PMC11574696

[6] Gupta SS Gupta SS Chawla R Gupta KS Bamrah PR Gokalani RA. Gestational diabetes mellitus—neonatal and maternal outcomes in women treated with insulin or diet: a propensity matched analysis. Diabetes Metab Syndr. (2024) 18(10):103145. 10.1016/j.dsx.2024.103145 39522430

[7] Shu X Yao M Li C Chen N Zhang Y Kang X Gestational diabetes mellitus and the longitudinal fetal growth trajectories in twin pregnancies. Twin Res Hum Genet. (2025):1–7. 10.1017/thg.2025.6 39949264

[8] Zhang Q Yuan X Luan X Lei T Li Y Chu W GLUT1 exacerbates trophoblast ferroptosis by modulating AMPK/ACC mediated lipid metabolism and promotes gestational diabetes mellitus associated fetal growth restriction. Mol Med. (2024) 30(1):257. 10.1186/s10020-024-01028-x 39707215 PMC11660491

[9] Fernandez-Alonso AM Monterrosa-Blanco A Monterrosa-Castro A Perez-Lopez FR. Gestational diabetes mellitus management according to ultrasound fetal growth versus strict glycemic treatment in singleton pregnancies: a systematic review and meta-analysis of clinical trials. J Obstet Gynaecol Res. (2024) 50(10):1759–70. 10.1111/jog.16059 39183485

[10] Karcz K Krolak-Olejnik B. Impact of gestational diabetes mellitus on fetal growth and nutritional status in newborns. Nutrients. (2024) 16(23). 10.3390/nu16234093 PMC11643953 39683486

[11] Chen T Qin Y Chen M Zhang Y Wang X Dong T Gestational diabetes mellitus is associated with the neonatal gut microbiota and metabolome. BMC Med. (2021) 19(1):120. 10.1186/s12916-021-01991-w 34039350 PMC8157751

[12] Okabe H Hashimoto K Yamada M Ono T Yaginuma K Kume Y Associations between fetal or infancy pet exposure and food allergies: the Japan environment and children’s study. PLoS One. (2023) 18(3):e0282725. 10.1371/journal.pone.0282725 36989214 PMC10057762

[13] Sweeting A Wong J Murphy HR Ross GP. A clinical update on gestational diabetes mellitus. Endocr Rev. (2022) 43(5):763–93. 10.1210/endrev/bnac003 35041752 PMC9512153

[14] Zhu Q Yang X Zhang Y Shan C Shi Z. Role of the gut microbiota in the increased infant body mass index induced by gestational diabetes mellitus. mSystems. (2022) 7(5):e0046522. 10.1128/msystems.00465-22 36154141 PMC9601173

[15] Zheng W Wang J Zhang K Liu C Zhang L Liang X Maternal and infant outcomes in women with and without gestational diabetes mellitus in the COVID-19 era in China: lessons learned. Front Endocrinol (Lausanne). (2022) 13:982493. 10.3389/fendo.2022.982493 36482992 PMC9723325

[16] Adjibade M Vigneron L Delvert R Adel-Patient K Divaret-Chauveau A Annesi-Maesano I Characteristics of infant formula consumed in the first months of life and allergy in the EDEN mother-child cohort. Matern Child Nutr. (2024) 20(4):e13673. 10.1111/mcn.13673 38786654 PMC11574648

[17] Suaini NHA Koh QY Toh JY Soriano VX Colega MT Riggioni C Maternal and infant dietary patterns are not related to food allergy risk in Singapore children: GUSTO cohort study. J Nutr. (2024) 154(7):2157–66. 10.1016/j.tjnut.2024.05.002 38740185 PMC11282467

[18] Sid Idris F Anis Shaikh H Vahora I Moparthi KP Al Rushaidi MT Muddam M Maternal diet and infant risk of eczema and food allergy: a systematic review. Cureus. (2023) 15(9):e45114. 10.7759/cureus.45114 37842462 PMC10569370

[19] Rossi G Cesca J Fong C Wallace A DComm PS Osuagwu UL Rural parents’ adherence to infant feeding guidelines to prevent allergy: a cross sectional study in New South Wales. BMC Public Health. (2023) 23(1):2458. 10.1186/s12889-023-17396-8 38066470 PMC10704778

[20] Dupont C Bocquet A Brancato S Chalumeau M Darmaun D de Luca A Cow’s milk-based infant formula supplements in breastfed infants and primary prevention of cow’s milk allergy: a commentary of the committee on nutrition of the French society of pediatrics. Arch Pediatr. (2023) 30(8):591–4. 10.1016/j.arcped.2023.07.005 37709607

[21] Hendrickx DM An R Boeren S Mutte SK, PRESTO study team, Lambert JM Assessment of infant outgrowth of cow’s milk allergy in relation to the faecal microbiome and metaproteome. Sci Rep. (2023) 13(1):12029. 10.1038/s41598-023-39260-w 37491408 PMC10368738

[22] Huang D Liang M Xu B Chen S Xiao Y Liu H The association of insufficient gestational weight gain in women with gestational diabetes mellitus with adverse infant outcomes: a case-control study. Front Public Health. (2023) 11:1054626. 10.3389/fpubh.2023.1054626 36908424 PMC9996046

[23] Lu L He L Hu J Li J. Association between very advanced maternal age women with gestational diabetes mellitus and the risks of adverse infant outcomes: a cohort study from the NVSS 2014–2019. BMC Pregnancy Childbirth. (2023) 23(1):158. 10.1186/s12884-023-05449-0 36899316 PMC9999489

[24] De Paepe E Van Gijseghem L De Spiegeleer M Cox E Vanhaecke L. A systematic review of metabolic alterations underlying IgE-mediated food allergy in children. Mol Nutr Food Res. (2021) 65(23):e2100536. 10.1002/mnfr.202100536 34648231

